# Discovery of Electrochemical Indicators upon Sarcoplasmic Meat Discoloration

**DOI:** 10.1021/jacs.4c09375

**Published:** 2024-10-30

**Authors:** Sandun Bogahawaththa Kasthuri Dias, Silan Bhandari, Sachinthani A. Devage, Jennifer A. Avery, Rishav Kumar, Ranjith Ramanathan, Sadagopan Krishnan

**Affiliations:** † Department of Chemistry, Oklahoma State University, Stillwater, Oklahoma 74078, United States; ‡ Department of Animal and Food Sciences, Oklahoma State University, Stillwater, Oklahoma 74078, United States

## Abstract

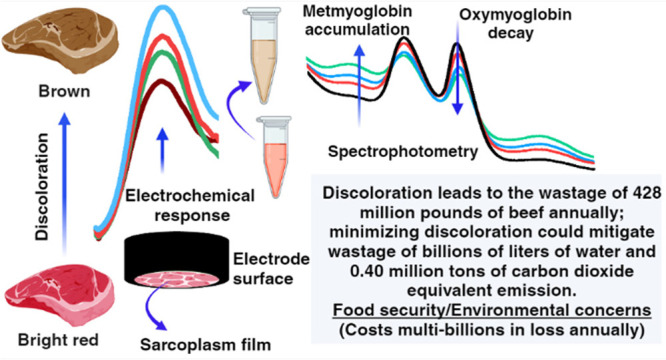

Meat discoloration is one of the challenges facing the food industry, which affects both quality and shelf life. In this report, we present our groundbreaking discovery of electrochemically probing specific redox peaks associated with meat discoloration and successfully monitor its delay when controlled biochemically with added antioxidants. We have validated the redox features by spectrophotometry measurements of the relative levels of oxymyoglobin, which gives meat its cherry red color, and metmyoglobin, which causes the meat to turn brown in relation to discoloration. The insights from this research open up new avenues for the development of innovative electroanalytical tools for studying meat color and quality. These new tools could potentially minimize nutritious beef waste, lessen the environmental burden associated with waste disposal, and reduce CO_2_ emissions linked to discoloration issues.

Meat is a rich source of protein, minerals, and vitamins and an essential component of the human diet.^1^ Meat color, an important quality attribute that influences purchasing decisions, is affected by various biochemical and environmental factors.^2,3^ Myoglobin, a protein that can exist in several redox states, is the primary determinant of meat color. The reduction of metmyoglobin and the subsequent formation of oxymyoglobin are crucial processes that give fresh beef its bright cherry red color. Conversely, deoxymyoglobin imparts a purplish hue to beef, while unreduced metmyoglobin can cause the meat to turn brown.^4−6^

Although various pre- and post-harvest factors can influence meat discoloration (microbial spoilage, lipid oxidation, antioxidant activity, and protein oxidation),^7^ understanding these color changes is crucial for maintaining the quality and appearance of meat products and ensuring consumer acceptance.^8^ For example, consumers exhibit a preference for beef with a bright cherry red color, which is associated with freshness and wholesomeness,^8^ and tend to discriminate against any variation from this color, leading to significant economic losses globally, food security issues, and environmental concerns.^9,10^ In the United States, the beef industry loses approximately $3.7 billion and discards 195 million kg of beef (equivalent to wasting 780 000 animals) annually due to discoloration. Addressing discoloration issues is not just about economic benefits but also about our responsibility toward the environment. It could save billions of gallons of water and tens of billions of megajoules of energy consumption while reducing carbon dioxide emissions by several tons.^11^

Among various analytical methods, such as spectrophotometry, mass spectrometry, and chromatography, which are utilized in studying meat muscle proteins and their proteomics,^12,13^ currently, no electrochemical techniques are available to monitor discoloration. At retail stores, a meat store employee monitors meat visually. If there is discoloration, stores try to sell it with a discount or discard it. Researchers use hand-held devices such as HunterLab MiniScan spectrophotometers to measure color.^14^ Another assay, “RedoxSys”, is a commercially available oxidative stress analyzer device that measures the oxidation–reduction potential as a homeostatic parameter but not meat discoloration.^15,16^

Prior literature work confirmed that reducing activity is an important factor influencing discoloration. The development of a tool to predict discoloration or to understand the reduction potential of meat is a crucial step in our understanding of meat discoloration. This research is significant as it could have a tremendous impact on minimizing the discarding of 400 million pounds of nutritious beef. Several studies have been conducted to understand the reason why meat discolors. In this research, a novel approach is utilized to measure redox activity by an electrochemical method, which offers unique advantages. These advantages include on-site simplicity (no laboratory or complex instrumentation settings required). The user-preferred features of these methods include their speed, portability (hand-held, lightweight devices), cost-effectiveness (inexpensive, readily available carbon electrodes), enhanced sensitivity (through selectively probing desired molecular- and atomic-level redox properties of target species), versatile throughput options (e.g., a 96-electrode plate similar to that used in a biological assay kit), and extrapolation of the present study incorporating low-cost cells and electrodes will facilitate the design of disposable devices.^17,18^

In this study, we build on these advantages and introduce a label-free, calibration-free, and user-friendly electroanalytical tool that can monitor meat discoloration in real time by displaying reliable redox peak indicators. The innovative results presented in this report regard both the fundamental redox properties of sarcoplasmic extracts, confirmed through spectrophotometry and the significance of translating this fundamental molecular property as a diagnostic tool useful for reducing the waste of nutritious beef.

Figure 1 illustrates the experimental design for our electrochemical meat discoloration approach. It integrates spectrophotometric confirmation via the estimation of oxymyoglobin and metmyoglobin levels using beef sarcoplasmic extract samples (Figures S1–S3 for the extract preparation and characterization). For the electrochemical probing of meat redox peaks, we employed square wave voltammetry, which offers good analytical sensitivity through an increased signal-to-background ratio.^19^

**Figure 1:**
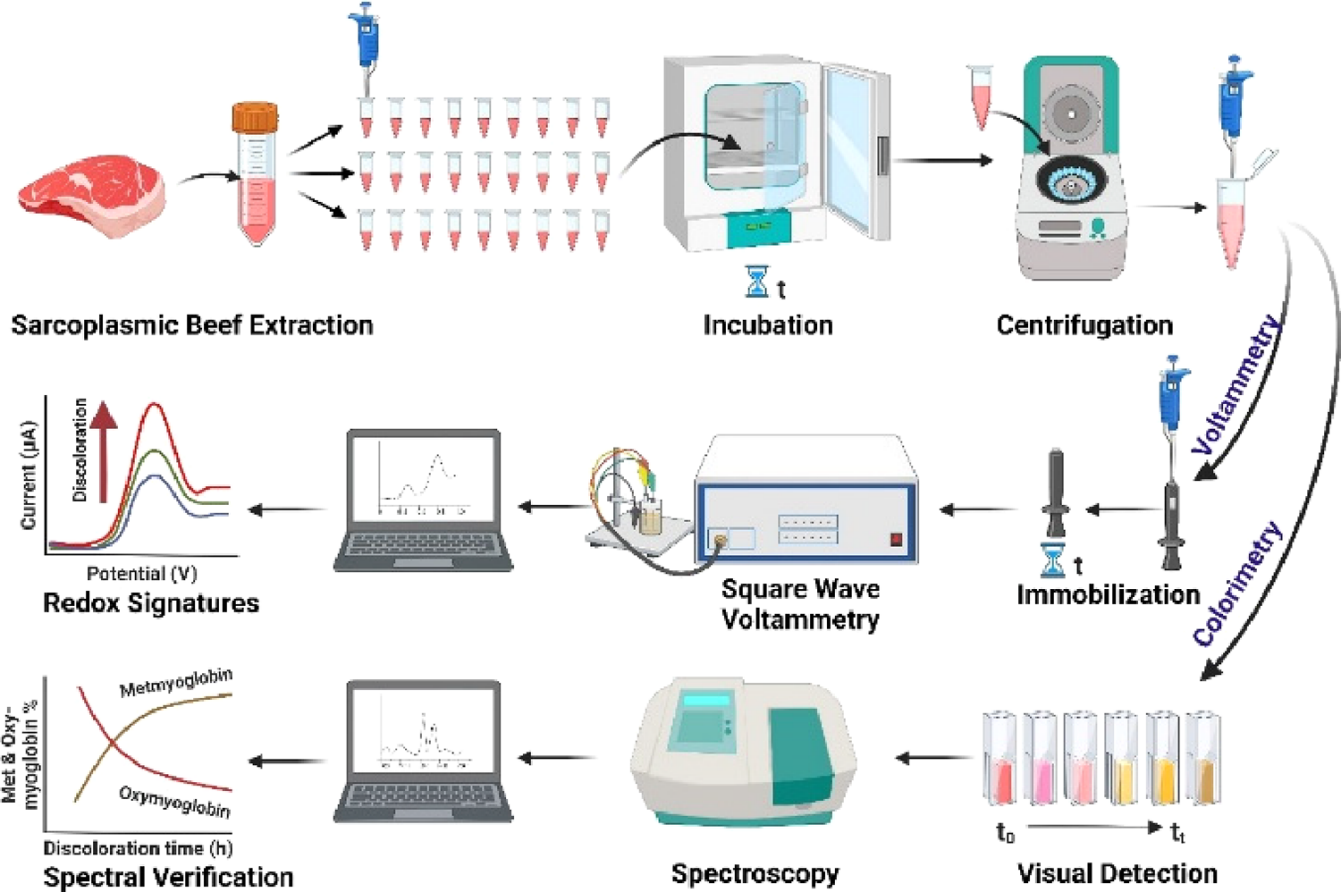
Schematic diagram depicting the research design for the electrochemical analysis of meat discoloration. First, beef sarcoplasmic extraction is achieved through differential centrifugation. Subsequentially, the extracted samples are incubated in aliquots for varying durations at 37 °C. After incubation, the samples are centrifuged, and a polished clean graphite disk electrode is coated with an aliquot (10 μL) of the meat extract. Then, a voltammetric scan is conducted to identify the redox peak indicators of meat discoloration. At the same time, spectrophotometric quantitation is achieved using the metmyoglobin (503 nm) and oxymyoglobin (582 nm) absorbance bands.^22,23^ Calculating the percent of metmyoglobin and oxymyoglobin relative to the absorbance values of each aliquot is an independent validation of the electrochemical redox indicators with meat discoloration.

By exploiting the inherent advantages of electrochemistry, in this study, we identified a strong correlation between the increase in current response and meat discoloration (Figure 2) at three potentials, 0.42 ± 0.02, 0.86 ± 0.02, and −0.260 ± 0.005 V vs Ag/AgCl (average ± SD for *N* = 5 replicates), at pH 5.6 (post-mortem meat pH), saturated oxygen, 37 °C (data plot is shown in Figure 2A, Figure 2B for visual depiction, and voltammograms in Figure 2C for peaks at +0.42 V and +0.86 V in the positive potential region, and Figure 2D for the peak at −0.26 V in the negative potential region). The observed electrochemical data correlated with the increase in metmyoglobin levels at 503 nm (indicating discoloration) and the decrease in oxymyoglobin levels at 582 nm (diminishing red color) measured spectrophotometrically, as shown in Figure 2E. The peaks in the positive potential region likely attribute to the protein and lipid oxidation during meat discoloration previously established biochemically,^4,20,21^ and the associated increase in oxidative currents with time.

**Figure 2:**
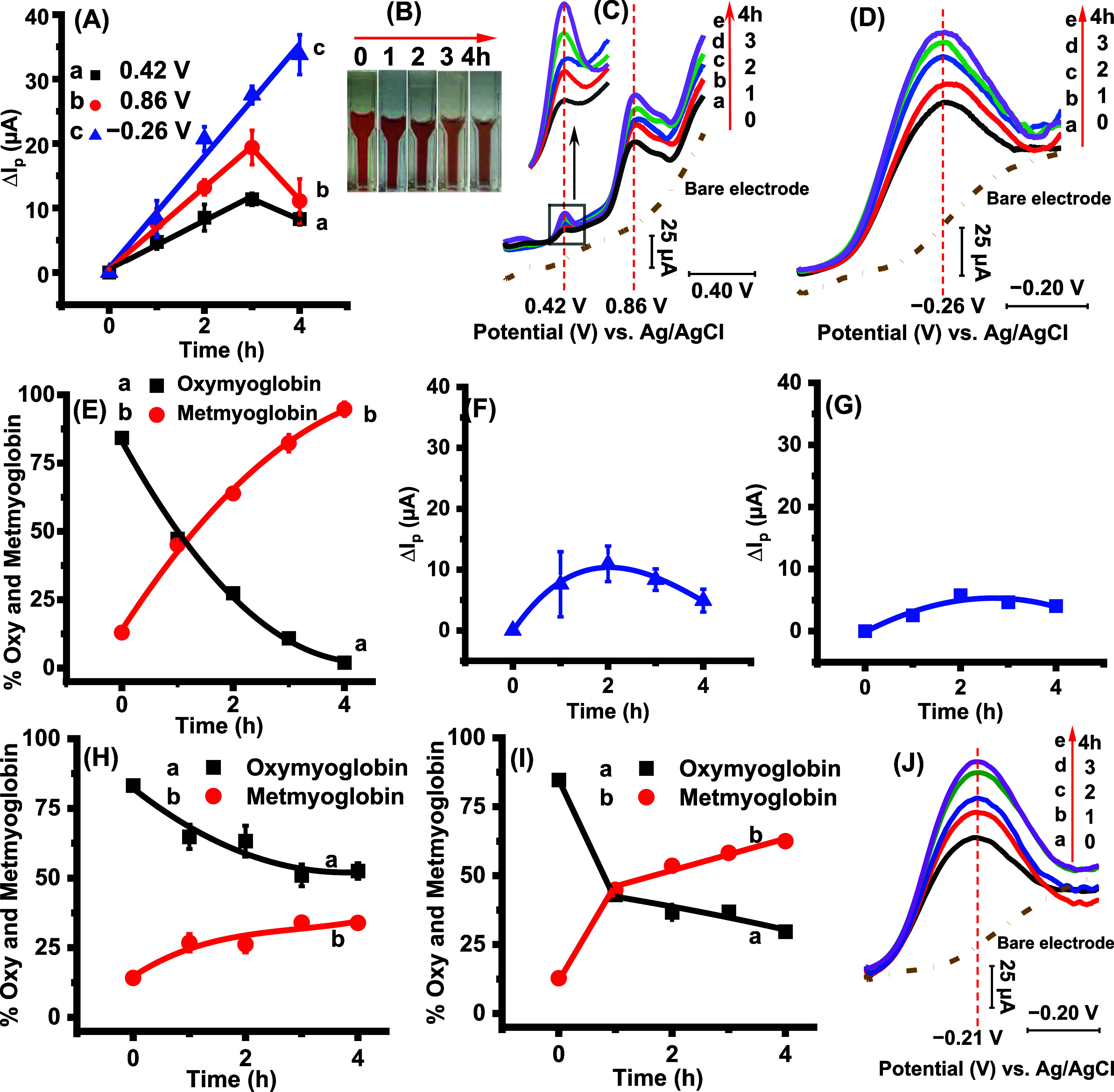
Electrochemical peaks reflecting meat discoloration at 0.42 ± 0.02 V, 0.86 ± 0.02 V, and −0.260 ± 0.005 V vs the Ag/AgCl reference at pH 5.6 (post-mortem meat pH) in saturated oxygen buffer: (A) The peak current increases with incubation time at 37 °C for the three potentials discovered in this study. Fresh electrodes were used for the positive and negative potential scans. (B) An image showing meat discoloration with incubation time at 37 °C. (C, D) The square wave voltammograms show an increase in peak current at the two positive potentials (panel (C)) and the negative potential (panel (D)), respectively. Polished bare electrodes in the absence of coated meat extract film do not show any peaks like the samples (broken lines). (E) The corresponding decrease in oxymyoglobin (red color of meat) and increase in metmyoglobin accumulation (discoloration) percentages with incubation time. The discoloration delay effect in the presence of added (F) 5 mM ascorbic acid (0.39 ± 0.01 V vs Ag/AgCl) and (G) 5 mM NADH (0.38 ± 0.01 V vs Ag/AgCl) showed a smaller current change with incubation time and the proportionately lower spectral estimation of oxy and metmyoglobin percentages with incubation time, as shown in panels (H) and (I), respectively (*N* = 5 replicates). (J) The voltammograms of purified beef myoglobin in the negative potential region at −0.214 ± 0.002 V (*N* = 5 replicates and four different batches of meat extracts were tested) show a similar current increase as the meat extract in panel (D).

The negative region incorporating the metmyoglobin reduction potential was observed at −0.26 V vs Ag/AgCl, and the peak increased with discoloration due to metmyoglobin accumulation. For the purified myoglobin-coated electrodes, the peak at −0.21 V vs Ag/AgCl (Figure 2J) increased with incubation time similar to the meat extract. The purified myoglobin peak is centered slightly more positively than the extract peak. The observed potential difference between the meat extract (−0.26 V vs Ag/AgCl) and isolated myoglobin (−0.21 V vs Ag/AgCl) is attributed to the presence of lipids and several other proteins in the sarcoplasmic extract likely causing a kinetic barrier, which influences the resulting redox potential to be slightly more negative. Moreover, we pursued experiments in both argon and oxygen atmospheres to delineate the direct electron transfer properties of the heme center of myoglobin with electrode surface in argon (noncatalytic) versus the oxygen atmosphere, with the latter leading to electrocatalytic myoglobin reduction.^24^ As shown in Figure S4 (panel (A) for the beef sarcoplasm extract and panel (B) for the purified beef myoglobin), under the argon atmosphere, the electrode probes the myoglobin heme center as a noncatalytic peak. In contrast, in the presence of oxygen, the electrocatalytically reduced ferryl-oxo complex formed from the accumulated metmyoglobin molecules upon discoloration is electrocatalytically probed with significantly higher currents than the anaerobic argon atmosphere.^25^ Both beef sarcoplasm extract (Figure 2D) and the purified beef myoglobin (Figure 2J) displayed similar trends in the negative potential region.

Furthermore, the positive potential region study of purified myoglobin displayed a prominent peak at 0.80 ± 0.02 V vs Ag/AgCl (*N* = 4 replicates) that also correlated with the meat extract response to discoloration (Figure S5A voltammogram), which was confirmed with the spectral quantitation of oxymyoglobin and metmyoglobin presented as Figure S5B. Thus, the contribution of myoglobin to meat discoloration has been successfully confirmed electrochemically.

The observed current increase at the described peak potentials is thus indicative of meat discoloration. Hence, factors that slow the discoloration process should be associated with minimal or negligible current change over time due to the delay in the discoloration. To test this hypothesis, we examined the influence of adding electron-donating cofactors, such as ascorbic acid (Figure 2F) and NADH (Figure 2G), which are known to delay meat discoloration, as they contribute to the preservation of the metmyoglobin reduction process and the ability to bind oxygen to form oxymyoglobin (which gives meat its red color). For both NADH and ascorbic acid, the peak response was slightly lower than the +0.42 V potential observed in the absence of them (Figures S6A and S6B), likely due to the differences in the electrolyte composition.

The delayed current changes, prominent at 3 and 4 h incubations, in the presence of NADH and ascorbic acid correlated with the relatively lower oxymyoglobin and metmyoglobin spectral absorbance changes (Figure 2H + ascorbic acid and Figure 2I + NADH) compared to the data presented in Figure 2E, which is in the absence of added NADH and ascorbic acid. Moreover, the electrochemical currents slightly decreased after 4 h incubation at +0.42 V and +0.86 V potentials (Figure 2A), but the trend in absorbance of the oxymyoglobin and metmyoglobin remained the same (Figure 2E). This is likely attributed to the higher sensitivity of the electrochemical redox signal changes in response to subtle changes at the meat extract film–electrode interface than the less-sensitive light-intensity ratio-based bulk solution absorbance phenomenon.

Taken together, the correlation between electrochemical and spectrophotometric oxymyoglobin and metmyoglobin estimates offers fundamental insights into the identified redox peaks in the positive and negative potential regions of the sarcoplasmic meat extract voltammograms upon meat discoloration. Our electrochemical approach is label-free because meat oxidation is directly measured upon discoloration and metmyoglobin accumulation. Moreover, our method is calibration-free because we can directly use discolored samples and refer to the corresponding fresh sample data as an internal reference to delineate differences in the current magnitudes with high accuracy.

The delay in the peak current increase upon the addition of electron donors such as NADH and ascorbic acid further supports the sensitivity of the electrochemical indicators in accurately probing the biochemically delayed discoloration process. In a broader sense, such characteristics will allow the testing of various practical conditions and the effects of novel reagents that delay meat discoloration through simple-to-use electrochemical technology as a valuable sensor tool in meat science. Our future studies will involve performing detailed separation and protein analysis and protein profiling upon discoloration to assign specific redox peaks to corresponding biochemical changes in meat. This underscores the broader significance and novelty of this first report on beef discoloration electrochemical studies. Our extended future studies will also address other related factors involved in meat discoloration monitored electrochemically.

In summary, we introduced an electroanalytical technique for meat science research with a specific focus on redox peak indicators and meat discoloration in this report. The identified redox indicators associated with sarcoplasmic beef discoloration provide valuable insights at a fundamental level. This knowledge can be applied to develop tools for measuring meat quality from a color perspective. Spectrophotometric confirmation of metmyoglobin accumulation and oxymyoglobin loss with discoloration and their delay in the presence of antioxidants supports the reliability of using the electrochemical method to probe meat discoloration. The demonstrated technique is label-free as the samples are directly analyzed without using indirect chemical or enzymatic assay indicators and a calibration-free tool as no reference standards are required for estimating the discoloration from the fresh sample property offering the method uniqueness compared with all prior analytical methods by specifically displaying meat discoloration redox peaks in meat samples.

## Supporting Information Available

Additional experimental details, materials, and methods; beef sarcoplasmic extract preparation procedure scheme (Figure S1), gel electrophoresis of extracted beef sarcoplasm (Figure S2); UV-vis spectral characterization of sarcoplasm extract (Figure S3); square wave voltammograms in argon and oxygen for beef sarcoplasm extract compared with isolated myoglobin (Figure S4); voltammograms of purified beef myoglobin in the positive potential region (Figure S5); and voltammograms in the presence of added ascorbic acid and NADH (Figure S6) (PDF)
