# Supplementary material for: Discovery of Electrochemical Indicators upon Sarcoplasmic Meat Discoloration
Source: J Am Chem Soc. 2024 Oct 30;146(45):30728–32. doi: 10.1021/jacs.4c09375 (PMC11565638; doi:10.1021/jacs.4c09375)
Supplement: Supplementary file 1 — ja4c09375_si_001.pdf [file ja4c09375_si_001.pdf]

Supporting Information

Discovery of Electrochemical Indicators Upon Sarcoplasmic Meat Discoloration

Sandun Bogahawaththa Kasthuri Dias,[a,‡] Silan Bhandari,[a, ‡] Sachinthani A. Devage,[a, ‡] Jennifer A. Avery,[a] Rishav Kumar,[b] Ranjith Ramanathan,[*,b] Sadagopan Krishnan[*, a]

[a]Department of Chemistry, Oklahoma State University, Stillwater, OK 74078, USA

[b]Department of Animal and Food Sciences, Oklahoma State University, Stillwater, OK 74078, USA

Experimental.

Methods.

Chemicals and Materials.

High-purity graphite (HPG) disc electrodes, silicon carbide (SiC, EXTEC CORP, CT, USA), P320 grit paper, monosodium hydrogen phosphate (NaH_2PO_4. H_2O), disodium hydrogen phosphate (Na_2HPO_4) and sodium chloride (NaCl) were purchased from Sigma–Aldrich (St. Louis, MO, USA). Deionized water was used to prepare the buffer solutions, and the pH of the buffer was adjusted to 5.6 using a pH meter (Fisher Scientific, Model: AB 15 Plus).

Beef Sarcoplasm Extraction.

The sarcoplasmic extract samples were prepared using previously described methods (**Figure S1**).[1] Briefly, ten grams of beef meat, which is visually free from fats and connective tissues, was homogenized with 30 mL of pH 5.6 phosphate buffer for 30 seconds. The homogenized meat sample was allowed to flow through double-layered cheesecloth into a 50 mL beaker. The filtrate was transferred to 1.5 mL Eppendorf tubes and centrifuged at 14000 × g for 5 minutes. After centrifugation, the supernatant was collected and filtered through another double-layered cheesecloth to obtain the clear sarcoplasm extract. The extract was incubated at 37 °C in a Benchmark H2200-HC incubator for 0 to 4 h. Before each measurement, beef sarcoplasm was centrifuged at 4000 revolutions/minute for 4 min by using an Eppendorf 5424 centrifuge. The initial measurement was used as a control and was performed without incubation (0 h sample).

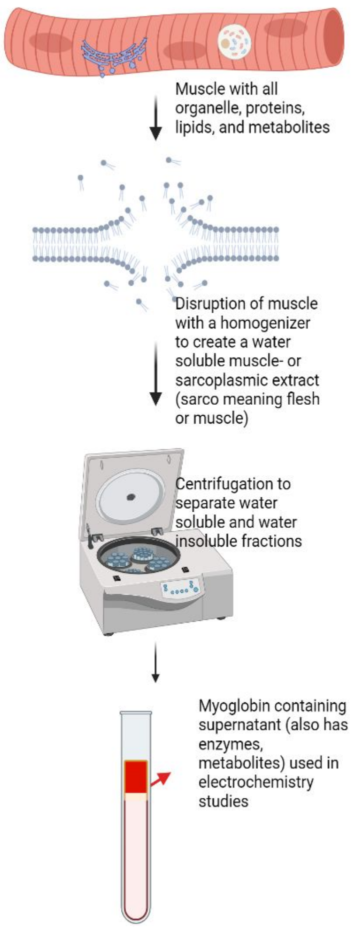

Figure S1. Outline of beef sarcoplasmic extract preparation procedure for the electrochemical and spectrometric studies.

Measuring Redox Potentials.

Electrochemical measurements were conducted (in saturated argon or oxygen conditions) using a CH potentiostat (Model CHI 6017) and a standard 3-electrode electrochemical cell equipped with an Ag/AgCl (3 M KCl) reference electrode, a Pt-wire counter electrode, and polished high-purity graphite-disk (HPG, geometric area 0.316 cm^2) working electrodes coated with meat extract. The HPG electrodes were polished on SiC-grit 320, sonicated for 10 seconds in ethanol and 10 seconds in water, washed with deionized water, and dried under ultrapure nitrogen to ensure a clean surface prior to coating with the meat extract.

The meat extract biofilm was prepared by placing an extract solution (10 µL, pH 5.6) on the freshly polished HPG electrodes for 15 min at room temperature (23 °C) to allow physisorption through electrostatic and other secondary interactions, followed by drying in air, thus resulting in a biofilm coating with a measurement time of 10 s (1 V range at a 0.1 Vs^{-1} scan rate in 10 s, fast measurements). For the purified beef myoglobin, a 1.3 mg/mL solution at pH 5.6 was prepared, and 10 µL of the myoglobin solution was coated on an electrode.

After the electrodes were rinsed in deionized water, analytically sensitive pulse square wave voltammetry was performed in a magnetically stirred (150 rpm) 6 mL phosphate buffer. Two separate ranges were studied, from 0 to +1.0 V and from 0 to –0.8 V, using new sample electrodes for each range. The aim was to determine the signature peak potentials associated with meat protein oxidation in the positive potential range and metmyoglobin accumulation in the negative heme center region. The square wave voltammetry conditions used were a 4-mV step height, a 25-mV pulse height, and a 15 Hz frequency.

Spectroscopic and Visual Detection.

Spectrophotometric determination was performed in a UV–visible spectrophotometer (Varian Cary 100 Bio, Varian Inc., CA) with sample extracts placed in standard spectroscopic cuvettes (1 cm × 1 cm × 4.5 cm) to measure the absorbance in the wavelength range of 400-700 nm. Three replicates of sarcoplasmic extract were used for each measurement, and a baseline was set using a pH 5.6 phosphate buffer solution. At each experimental time point (0 to 4 h), the electrochemically monitored onset and progress of discoloration were used to estimate the corresponding oxy- and metmyoglobin percentage variations by measuring their absorbance values using the spectrophotometer. The visual images of the sarcoplasmic extract showing a discoloration pattern at each incubation point were collected using an iPhone 14 Pro Max 17.3.1 phone.

Protein gel electrophoresis characterization of beef sarcoplasm extract (**Figure S2**: Lane 1 – MW marker standards, Lane 2 – Purified bovine myoglobin, Lane 3 – Beef sarcoplasm extract). Samples for sodium dodecyl sulfate-polyacrylamide gel electrophoresis (SDS-PAGE) were prepared using a sample buffer consisting of 2-mercaptoethanol and 4x Laemmli sample buffer in a 1:9 ratio. The sarcoplasmic proteins (protein concentration 1 µg/µL, 16 µL was loaded onto the gel) were mixed with the sample buffer in a 3:1 ratio and heated at 95°C for 5 minutes. The 14% hand-cast gradient gels were prepared using Mini-PROTEAN Casting Plates (dimensions 10.0 x 8.0 cm), and the electrophoresis run was performed in a Mini-PROTEAN® Tetra Vertical Electrophoresis Cell (Bio-Rad Laboratories, Hercules, CA). Gels were stained with Coomassie Blue G250. Destained gel images were acquired using the Biorad Gel Doc EQ Imaging system (Bio-Rad Laboratories, Hercules, CA) with a white light conversion screen. The images were analyzed with Image Lab 6.0.1, © 2017 Software (Bio-Rad Laboratories, Hercules, CA) to determine the defined bands. Protein molecular weights were identified by comparison with a known molecular weight standard (Precision Plus Protein™ All Blue Prestained Protein Standards, 8 µL was loaded onto the gel, Bio-Rad Laboratories, Hercules, CA).

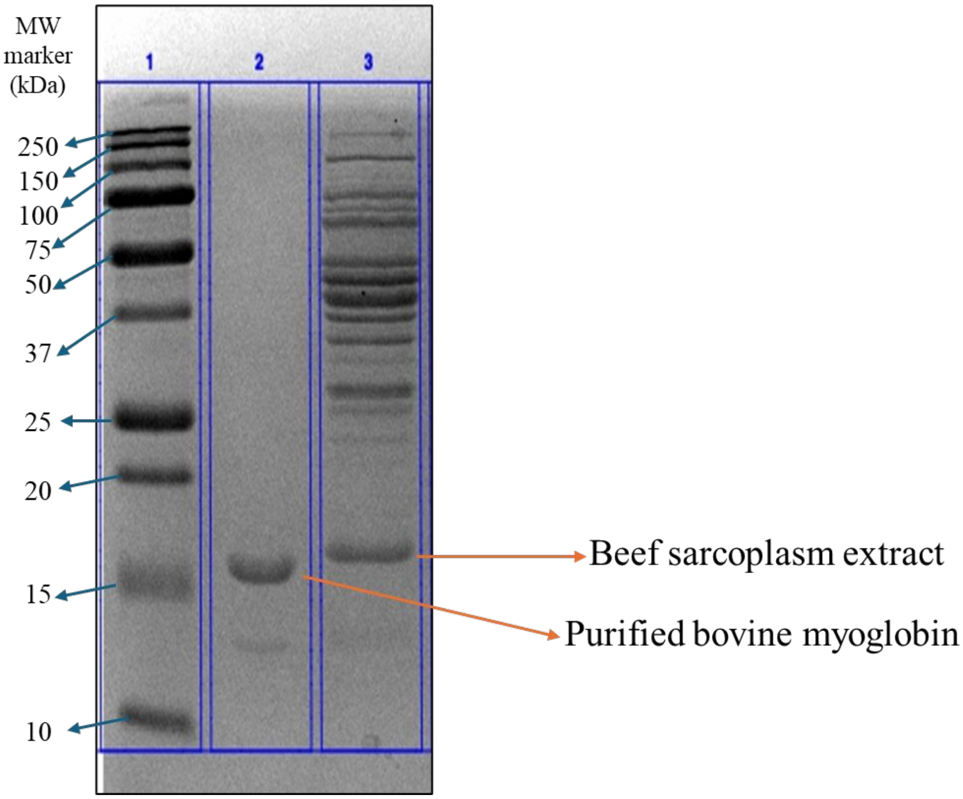

Figure S2. SDS-PAGE analysis of the extracted beef sarcoplasm.

Sarcoplasm contains several proteins. However, myoglobin is the primary heme protein that imparts a red color to meat. In the SDS-PAGE gel, several protein bands were observed, as expected in a sarcoplasmic fraction. Lane 1 is the molecular weight marker standard, Lane 2 is the purified beef myoglobin (myoglobin purification protocol is included below), and Lane 3 contains different bands from the sarcoplasm extract, including the myoglobin band migrating close to the purified isolated bovine myoglobin. The gel electrophoresis characterization combined with the spectrophotometry confirmed the presence of and access to myoglobin in the extract.

Purification of myoglobin.

Myoglobin was purified via ammonium sulfate precipitation and gel filtration chromatography, as per the procedure published by Faustman and Phillips.[2] Briefly, beef cardiac muscle devoid of fat and connective tissues was homogenized in buffer (10 mM Tris–HCl, 1 mM EDTA, pH 8.0, 4 °C) and centrifuged at 5000 ×g for 10 min. The supernatant was brought to 70% ammonium sulfate saturation, and the resulting solution was stirred for 1 h at 4 °C and later centrifuged at 18,000 ×g for 20 min. The resulting supernatant was saturated with ammonium sulfate (100%) and

centrifuged at 20,000 ×g for 1 h. The precipitate was resuspended in homogenization buffer and dialyzed (3 volumes) against 10 mM Tris–HCl, 1 mM EDTA, at pH 8.0, 4 °C for 24 h. Myoglobin was separated from hemoglobin using a Sephacryl 200-HR gel filtration column (2.5×100 cm). The elution buffer contained 5 mM Tris–HCl and 1 mM EDTA at pH 8.0, and the flow rate was 60 mL/h.

Isolated myoglobin solution was passed through a PD-10 column pre-calibrated with phosphate buffer, pH 5.6; 120 mM potassium chloride, 5 mM KH_2PO_4, and 30 mM maleic acid. Myoglobin was reduced by sodium hydrosulfite (also called sodium dithionite)-mediated reduction (0.1 mg sodium hydrosulfite to 1 mg myoglobin). Residual hydrosulfite was removed using a PD-10 column, and reduced myoglobin was converted to oxymyoglobin by bubbling with oxygen. Myoglobin concentration was confirmed using absorbance at 525 nm ($A_{525\ nm}$ = 7.6 mM^{-1} cm^{-1}).

Addition of dithionite to confirm myoglobin redox state.

Dithionate is a reducing agent commonly used in myoglobin preparation. Dithionite will convert metmyoglobin or oxymyoglobin to deoxymyoglobin. Metmyoglobin has a wavelength maximum of 503 nm, oxymyoglobin has a wavelength maximum of 581 nm, and deoxymyoglobin has a wavelength maximum of 557 nm. Sarcoplasm was prepared according to the methodology discussed in the manuscript. The addition of dithionite converted the peaks to deoxymyoglobin, further indicating that the color pigment was myoglobin (**Figure S3**).

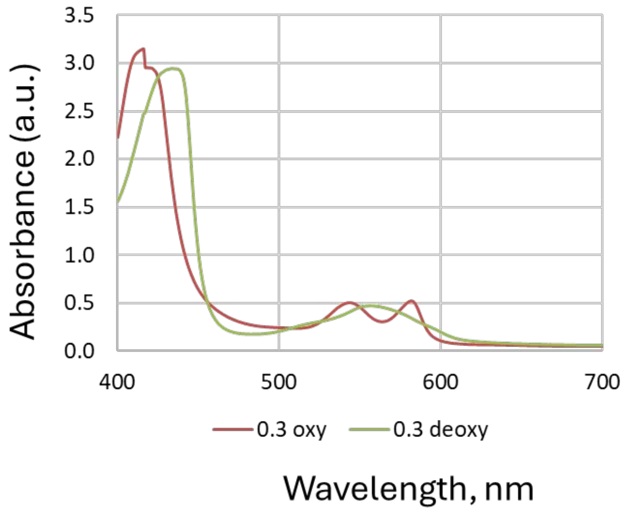

Figure S3. UV-Vis spectral characterization of myoglobin bands in the prepared sarcoplasm extract and upon reduction by sodium dithionite.

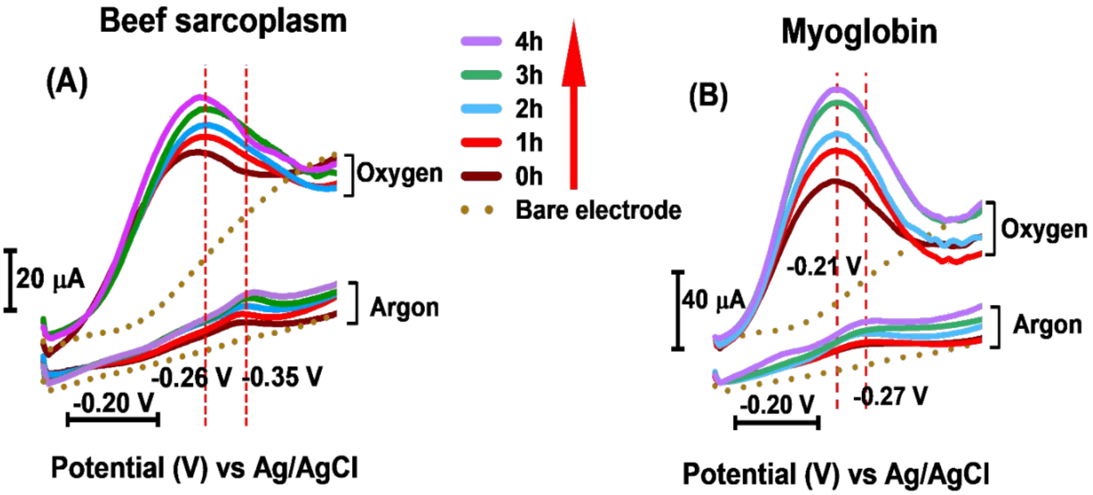

Figure S4. Electrochemical peaks from the square wave voltammograms in argon vs. oxygen atmosphere at pH 5.6 under 37 ° C: **(A)** beef sarcoplasm extract, and **(B)** purified beef myoglobin. Under the argon atmosphere, we are probing the myoglobin heme center as a non-catalytic peak. In contrast, in the presence of oxygen, the sensor probes the electrocatalytically reduced ferryl-oxo complex formed from the accumulated metmyoglobin molecules upon discoloration. Both the beef sarcoplasm extract and the purified beef myoglobin display similar trends in the negative potential region. Thus, the contribution of myoglobin to meat discoloration is monitored electrochemically.

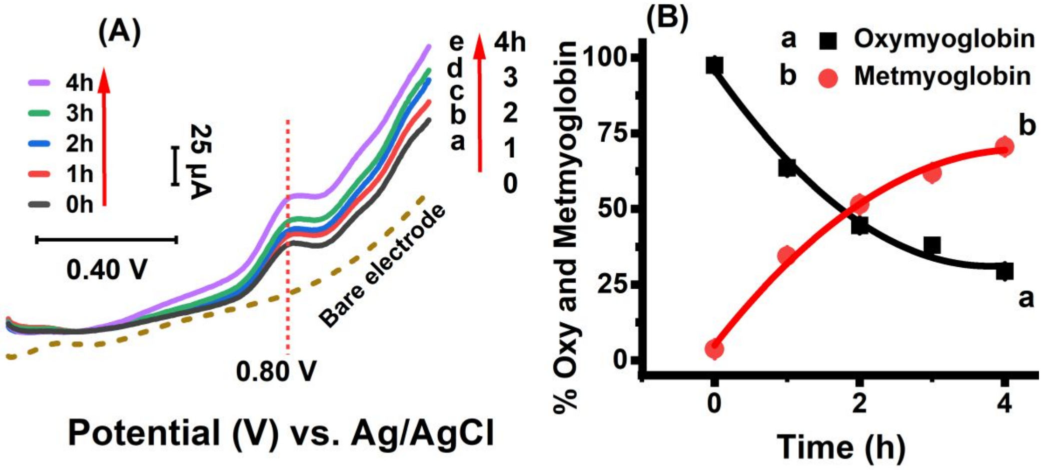

Figure S5. (A) The voltammograms of purified beef myoglobin in the positive potential region at 0.80±0.02 V vs. Ag/AgCl (N = 4 replicates) showed an increase in peak currents from 0 to 4 h incubation time at pH 5.6 and saturated oxygen buffer. (B) The UV-spectral measurement (N=4, pH 5.6 buffer as a blank solution) of purified beef myoglobin showed growth of metmyoglobin content and a decline in oxymyoglobin content accordingly with an incubation period (0 to 4 h).

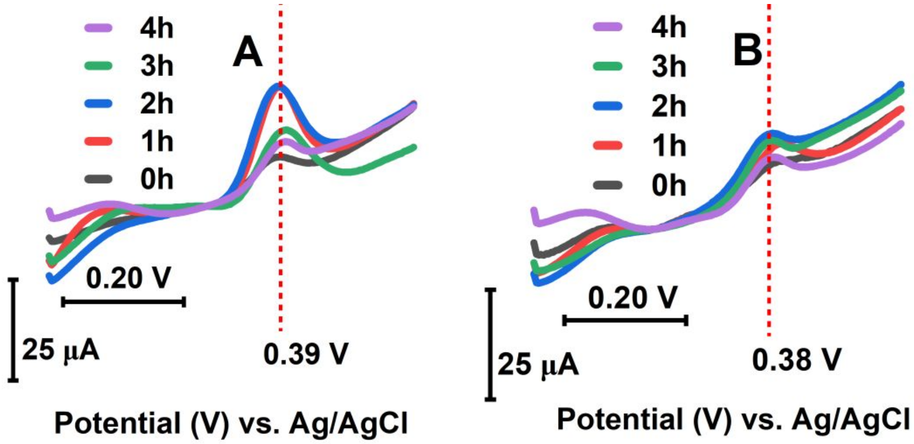

Figure S6. Square wave voltammograms in the presence of added electron-donating cofactors (5 mM each) into the buffer solution and scanned for the beef sarcoplasm extract electrode film: **(A)** Ascorbic acid at 0.39 ±0.01 V and **(B)** NADH at 0.38 ±0.01 V.

1. Ramanathan, R.; Konda, M. K.; Mancini, R. A.; Faustman, C. Species-Specific Effects of Sarcoplasmic Extracts on Lipid Oxidation *in vitro*. *J. Food Sci.* **2009**, *74*, C73–C77.
2. Faustman, C.; Phillips, A. L. In: R. E. Wrolstad, Ed., Current Protocols in Food Analytical Chemistry, Wiley & Sons, Inc., New York, **2001**, pp. F3.3.1-F3.3.13.
